# Supplementary material for: Are carbon emissions trading and green financial instruments synergistic? -Comprehensive quantitative research based on content analysis
Source: PLoS One. 2024 Mar 7;19(3):e0298601. doi: 10.1371/journal.pone.0298601 (PMC10919601; doi:10.1371/journal.pone.0298601)
Supplement: S1 Data — (ZIP) [file pone.0298601.s001.zip › Data/地级市Z_F工作报告.zip!/░▓╗╒/╨√│╟╩╨/└┤╫╘╣1⁄2╓┌║┼í╛┬φ┐╦╩2╛▌═°í┐_2012.docx]

2012年1月11日在宣城市第三届人民代表大会第二次会议上

市人民政府市长	虞爱华

2011年工作回顾

　　2011年，全市人民在市委的坚强领导下，坚持在发展中谋转型、在转型中促发展，全市经济社会继续保持了十分强劲的发展势头，主要经济指标增幅实现“两个超过”：超过年初人代会确定的目标，超过全省平均水平。

　　经济总量迈上新台阶。初步预计，完成生产总值670亿元左右，接近2000-2003年四年总量，增长14%左右。规模工业增加值340亿元左右，相当于2000-2007年八年总量，增长25%左右。财政收入118.6亿元，超过2000-2006年七年总量，增长40%，其中地方财政收入68.4亿元，超过2000-2006年七年总量，增长37.2%，财政收入占生产总值的比重达到17.7%左右。固定资产投资635亿元左右，超过2000-2006年七年总量，增长30%以上。社会消费品零售总额230亿元左右，接近2000-2003年四年总量，增长18%左右。进出口总额9亿美元左右，接近2000-2006年七年总量，增长31%左右，其中,出口7.7亿美元左右，超过2000-2006年七年总量，增长31.5%左右。实际到位省外资金超过1100亿元，超过2000-2009年十年总量，增长70%以上。实际利用外资3亿美元以上，相当于2000-2006年七年总量，增长38%左右。城市居民人均可支配收入17800元左右，农民人均纯收入8000元左右，分别增长18%左右和20%左右，城乡居民收入增幅均为建市以来最高。

　　实现了三大愿望。期盼几十年的水阳江下游“开卡”工程获得国家发改委批准，并正式动工。宣城设立海关、出入境检验检疫局已经中央相关部门正式批准。合工大宣城校区获教育部正式批准并开工建设，不仅圆了宣城人民二十多年来的本科院校梦，也使我市成为继合肥之后全省第二个拥有“211工程”大学的城市。

一年来，我们主要做了以下六个方面的工作：

（一）坚持不懈调整结构，产业转型明显加快。

　　工业优化升级持续推进。工业化率达到44%左右，较上年提高5.6个百分点。新增规模以上工业企业109家，总数达到689家，居全省第六位。工业经济效益综合指数达到322，创历史新高。完成工业投资330亿元左右，增长39.5%左右。积极培育战略性新兴产业，实现产值122.6亿元，增长70.2%。高新技术产业发展明显提速。实现总产值280亿元，增加值82亿元，分别增长66%和34%。传统优势产业稳步提升。

　　服务业发展势头强劲。服务业增加值220亿元左右，增长10.5%左右，其中交通运输业、批发零售业增加值分别增长8.5%和7.8%左右。市本级服务业重点项目26个，总投资超过200亿元。文化产业快速推进，15个项目进入省"861"项目库，8个项目获国家和省专项资金扶持，4个企业跻身省第三批文化产业示范基地。全年国内外游客接待量达到870万人次，增长35.5%；实现旅游总收入61.3亿元，增长39%，占生产总值的9%左右。

　　现代农业不断升级。宣州洪林现代农业示范区进入省级示范区行列，宁国南山食品产业园成为首批国家农业产业化示范基地。新增2户国家级农业产业化龙头企业，总量达到6户。新增省级农民专业合作示范社10个，位居全省首位。新增国家级标准化畜禽养殖示范小区2个、农业部地理标志认证产品5个。宁国、绩溪被认定为全国休闲农业与乡村旅游示范县。郎溪成为全省唯一的城乡一体化综合配套改革试点县。

（二）坚定不移招商引资，承接产业转移成效显著。

　　在皖江城市带承接产业转移示范区考核中名列第三。市开发区强力推进西扩区域“十路一桥”工程建设，“三横三纵”主次干道框架已经拉开。郎溪开发区跻身“中国最具发展潜力开发区100强、最佳投资服务环境开发区”行列。宁国开发区获得省创新型园区称号。广德、宁国分别被评为全省优势产业十强县和投资环境十佳县。项目体量进一步扩大，新签亿元以上省外投资项目257个，其中5亿元以上项目45个，10亿元以上项目18个。大力推进与央企和知名民营企业的合作，对接项目已开工224个，完成投资近300亿元。

（三）多方筹资加快基础设施建设，城乡面貌持续改观。

　　市本级完成城市建设投资110亿元，其中房地产投资48亿元，新开工和续建项目91个。交通建设全年完成投资28.9亿元。城东联圩和溪口枢纽除险加固工程加速推进，41座水库除险加固工程全面竣工，农村饮水安全工程全面完工，解决了18万人饮水安全问题。全市城镇化率达到45%左右，比上年提高近2个百分点。扎实开展“美丽和谐乡村”建设，稳步推进村庄整治、危房改造和清洁工程。泾县、绩溪成为省级村庄整治工作试点。创建省级园林城市通过验收。创建国家生态市和国家环保模范市工作全面启动，宁国、绩溪申报国家级生态县（市）通过环保部验收。

（四）千方百计化解难题，促进经济快速发展。

　　出台了鼓励企业上市政策意见，直接融资总额超过40亿元，达到历史最高水平。司尔特首发上市，江南化工实现定向增发，中鼎股份发行可转债，市国投公司首次获准发行10亿元公司债券。新开业金融机构18家。积极开展政银企对接合作，签约项目数、贷款数均超过上年水平。全年银行业金融机构新增贷款再次超百亿元。小额贷款公司累放贷款35亿元。融资性担保机构累计担保贷款40多亿元。整理清收闲置土地，加大用地报批力度，已经累计获得国家和省批准用地面积18565亩。

（五）多措并举改善民生，社会事业全面进步。

　　投入21.2亿元，全面完成33项民生工程。保障性住房建设工作位居全省前列。多渠道开发就业岗位，新增城镇就业近8万人，下岗失业人员再就业2万余人，登记失业率3.48%。超额完成五项社会保险扩面征缴任务，在全省率先实现城乡居民养老保险制度全覆盖。城乡低保和农村五保供养标准得到提高。城乡医疗救助“一站式”服务平台开通运行。荣获“全国文化体制改革先进地区”称号。宣州、宁国、泾县荣获“全国科技进步先进县”称号。完成68所义务教育阶段学校标准化建设，校安工程、留守儿童之家和校外活动室年度建设任务全部完成。各项人口指标继续位于全省前列，人口性别比持续保持正常，人口自然增长率控制在3‰以下。

（六）创新理念抓社会管理，社会局面和谐稳定。

　　扎实开展“四级书记大走访”和“千名干部下基层”活动，深入开展网络“政民互动”在线访谈活动，组织开展“百案攻坚”。在荣获全国“五五普法先进集体”的基础上，全面启动实施“六五”普法规划。7个县（市、区）全部成为省级平安县（市、区）。56个“难点村”治理任务全部完成，“阳光村务工程”通过省级达标验收。开展了打击违法生产经营建设行为、重点行业领域隐患排查整治行动和“清剿火患”战役，累计依法关闭煤矿、非煤矿山、危险化学品和烟花爆竹企业33家，安全生产态势平稳。

2012年发展目标和重点工作

　　今年政府工作的总体要求是：坚持科学发展主题和转型发展主线，把握“稳中求进、进中求快、快中求好”这一重要原则，把保增长作为首要任务，把改革创新作为强大动力，把保障改善民生作为根本目的，为建设经济强市、文化强市、生态强市奠定更加坚实的基础。

　　主要目标是：生产总值增长14%左右；规模工业增加值增长22%以上；财政收入增长20%以上；固定资产投资增长25%以上；社会消费品零售总额增长18%左右；外贸进出口总额增长25%以上，城乡居民收入增长15%以上。确保完成省定节能减排任务。

重点抓好以下八个方面的工作：

（一）着力推进示范区建设，承接产业转移要有新突破。

　　积极推进市开发区争创国家级开发区工作，加快落实市开发区世行贷款项目。壮大汽车及零部件、新型建材、LED等主导产业，发展现代装备制造、新能源、节能环保、生物医药等新兴产业。市开发区财政收入要力争突破8亿元。确保市本级华明飞彩、依维光电、金达利液压、鑫鸿汽车模具、中鼎产业园和宣酒工业园等项目建成投产，加快推进郎溪鸿泰特种钢、广德汽摩配产业园等项目建设。增强招大引强力度，确保实际到位省外资金、实际利用外资分别增长25%以上。

（二）着力加快转型升级步伐，壮大工业经济要有新进展。

　　统筹规划新兴产业发展基地，积极申报省级高新技术产业园，努力打造新兴产业集聚区。开工建设南漪湖风力发电、市区生活垃圾焚烧发电等项目。加快宣州博瑞特节能装备、郎溪特种设备等产业园建设。完成市科技产业服务中心一期工程建设。争取新增“科技路路通”创新服务站2个、省级工程技术研究中心3家以上、省级企业技术中心5家以上，新增高新技术企业15家以上。确保广德通用汽车研发中心建成运营。积极开展技术创新，实施省级以上创新计划项目50个。力争全年高新技术产业产值达320亿元、增加值100亿元左右。力争新创中国驰名商标2个和省名牌产品10个、著名商标38个。加快国家级文房四宝产品质量监督检验中心建设，确保宁国耐磨材料、泾县电机、绩溪不锈钢3个省级检验检测中心投入使用。确保宣城海螺二期、宣城电厂二期等项目开工建设。实施中小企业促进工程，力争培育亿元工业企业200家以上。鼓励支持企业开拓外贸市场，加强出口成长性企业的孵化培育，力争25家企业进出口过千万美元。组织实施40个重点节能改造项目，突出抓好32户重点耗能企业的节能降耗工作。加快国家生态市和国家环保模范市创建步伐。

（三）着力提升现代农业水平，新农村建设要有新举措。

　　深入实施农业产业化"262"工程，加快现代农业示范区和农产品加工园区建设。加快市粮食储备库建设，开工建设皖东南粮食批发市场。抓好立大禽业熟食加工和荣达禽业蛋品加工等项目建设，力争农产品加工产值超过400亿元。发展壮大烟叶、茶叶、油茶、中药材等特色高效农业。深入实施畜牧业升级计划和竹林丰产增效、水产跨越和农机化推进三项工程。加强“菜篮子”基地建设，通过2至3年的努力，确保市区蔬菜基本实现自给。认真开展宣州区农村房屋发证与抵押流转改革试点、郎溪县城乡一体化综合配套改革试点和宁国市统筹城乡土地使用制度综合改革试点。积极稳妥推进土地承包经营权流转，引导成立土地流转股份合作社。推进林权抵押贷款和森林保险工作，加快林权管理信息和森林资产评估、收储、担保、交易平台建设。继续实施“百村十镇示范工程”，深入推进全市新农村10条示范带建设，新增50个美丽和谐村庄，继续打造10个文化特色村。

（四）着力强化载体建设，服务业发展要有新成效。

　　推进中国茶府、百汇商贸和农副、农资产品批发市场等项目建设，纺织服装批发市场、联鑫家电市场、农机市场、国际竹艺商贸城、冷链物流、亚夏汽车财富广场等项目都要开工建设，力争新增限额以上企业100家。加快国购广场、星隆国际广场建设。开工建设市区大润发超市。加强城区菜市场标准化建设。开展“万村千乡市场工程”信息化改造试点。加快“新网工程”和再生资源回收利用交易市场建设。积极申报全国再生资源回收利用体系试点城市。力争引进招商银行、民生银行等金融机构，继续组建新的村镇银行，做好小贷公司、融资性担保公司监管工作。引导民间合法借贷，及时处置和打击非法集资行为，清理整顿非融资性担保机构。深入推进金融产品创新，完善社会信用体系建设，优化金融生态环境。积极引进风险投资，培育上市后备资源，抓好广信农化、黄山胶囊、五星食品、凤形耐磨、聚隆集团等企业上市工作。筹建市旅游发展投资公司。完善敬亭山基础设施，启动江南诗山文化园项目。开展龙川、大云岭红色景区5A创建和龙川申报国家级风景名胜区工作。加快扬子鳄自然保护区等项目建设。启动南漪湖总部经济园区和生态旅游度假区项目。加大文房四宝等特色旅游商品的研发。加快敬亭湖宾馆、万豪酒店等建设进度。同时，建立健全房地产市场体系和合理的住房供应体系，推进房地产业健康规范发展。

（五）着力加强基础设施建设，城镇化水平要有新提升。

　　提高规划水平，强化规划引导作用，加强规划执行的监管。确保市本级完成城市建设投资再超百亿元。继续推进城区“二环八射”路网建设。重点建设水阳江大道闭合段和宝城路改造。续建和新建环城大道等41条道路和28个市政配套项目。做好环城大道8标段、阳德路改造等9个项目前期工作。力争合工大宣城校区一期投入使用。完成宣中新校区主体工程。推进市体育中心、图书馆、规划馆和海关、出入境检验检疫局项目建设。做好市博物馆新馆、宣城大剧院、科技馆、美术馆、档案馆新馆等项目前期工作。完成仁和时代广场建设。启动彩金湖政务新区项目。继续推进“一山一河一湖多园”工程。建成敬亭湖公园、宣广高速宣城东出口公园。完成扬子鳄湖（暂定名）西湖景区主体工程，开工建设东湖景区及宛溪河综合整治二期工程。加快新港生态湖建设。整治泥河、道叉河。积极推进北门等棚户区改造，加快建设夏渡新城、桂花园、彩虹小区、付山新村等12个保障性住房和拆迁安置区工程。有序开展石油供应网点和城市燃气管网建设。同时，注重城管规范化、长效化和精细化，完善“数字城管”平台。健全禁违查违拆违联动机制，努力遏制违法建设。完成芜雁高速和宣广高速宣城东、西出入口改扩建工程。加快宣宁、宁绩、宁千高速建设。力争开工建设溧广高速。做好广宁、绩溪至谭家桥高速和鸿门至黄山连接线等项目前期工作。完成老318祠界路改建并通车，完成宣狸快速通道、梅岭隧道连接线、绩溪至长安段改建等主体工程。完成芜申运河宣州段建设，加快定埠段建设。力争开工建设市汽车客运总站。全力配合推进合福高铁宣城段建设，积极配合做好商杭高铁、皖赣铁路扩能改造等项目前期工作。强力推进水阳江下游“开卡”工程建设。继续加快城东联圩和水库除险加固工作。启动双桥联圩排涝泵站和堤防应急加固除险工程。推进青弋江灌区续建配套、港口湾灌区和郎源水库扩容、汤村水库、粮长门水库等工程前期工作，启动水阳江中游、新老郎川河综合治理等项目前期工作。继续推进农村安全饮水工程建设。认真做好电网“十二五”规划滚动调整和对接工作，启动市电力专项规划修编工作。建成投运110千伏城市中心变、赤滩变等5项工程，积极推进220千伏太平变、军塘变等11项工程。加快特高压主干电网建设和农网改造升级。加大通信设施的建设与保护，推动以3G为代表的现代通信技术发展和信息化产品运用，促进“无线城市”建设。

（六）着力发展文化产业，文化发展繁荣要有新气象。

　　力争跻身省级文化产业示范区。完善扶持政策，积极引进文化产业项目。加快建设夏渡文化创意产业园区，推进中国书画交易中心、中国工艺礼品城等项目建设，力争徽文化展示基地、动漫衍生品交易基地和少儿科普产业基地等项目开工建设。积极谋划广告传媒产业基地项目。坚持以工程推动文化事业的繁荣。继续实施文化惠民工程，加快重点公共文化设施建设。启动广德太极洞国家文化和自然遗产保护设施建设项目，实施绩溪历史文化街区保护设施建设项目。推进谢朓楼重建工程。加强文艺精品创作，积极做好电视剧《文房四宝》拍摄工作。坚持以改革激发文化活力的增强。培育新型文化市场主体，加快宣城报业集团、广电集团和市文化产业投资公司组建工作。推进市广电文化产业中心建设。支持中国宣纸集团、皖南海峰印刷包装公司等企业做大做强。

（七）着力保障改善民生，社会事业要有新发展。

　　继续加大投入，确保完成省定33项民生工程建设任务。落实和完善应对价格异动应急预案和工作机制，设立价格调节基金，加强民生商品价格监管。全面实施居民收入倍增规划。实施创业富民工程，深化省级“创业型城市”创建活动。建立健全职工工资正常增长和支付保障机制。推行企业工资集体协商制度，逐步提高最低工资标准。积极发展农村非农产业，扩大农村产权制度改革试点，构建农民增收多元支撑体系。落实和完善强农惠农政策，加强农民负担监管。扎实做好新阶段扶贫开发工作。继续加强就业和社会保障工作。实施更加积极的就业政策，加强就业培训，重点抓好城镇就业困难人员、高校毕业生和农民工就业，大力促进农村劳动力就近就地就业。加快建立统一规范的人力资源市场，加强人才引进和使用。扎实做好社会保险扩面提标工作，进一步完善城乡居民养老保险制度，促进社会保障体系更加健全，基金运行更加安全有效。完善城乡医疗救助“一站式”平台建设。确保市社会福利中心建成使用。各县（市）至少新建或改扩建1所社会（儿童）福利中心。支持社会化护理院建设。加强农村敬老院建设，提高“五保”集中供养率。加快市残疾人康复中心建设。加速推进学校布局规划和标准化建设，促进学前教育和义务教育均衡发展。力争新改扩建幼儿园40所，学前三年适龄幼儿毛入园率达到65%。加快发展职业教育，推进旌德和众高等职业教育产业园一期工程和宣城工业学校二期项目。继续深入开展全民健身活动。开展县级公立医院改革试点。深化卫生服务管理体制和运行机制改革，加强农村和社区卫生服务体系建设。完善重大疾病防控和公共卫生应急体系，提高医疗质量和服务水平。加快宣城中心医院、市精神病医院等项目建设。制定大中专毕业生到乡村卫生院（室）工作的激励政策。深化全国人口和计划生育综合改革示范市建设，确保各项指标继续保持全省领先水平。制定并实施好新一轮妇女儿童发展规划。加强统计基层基础工作。推进老龄事业和红十字、慈善事业健康发展。做好民族宗教、外事侨务、对台、档案、地方志、防震减灾、气象、烟草专卖等各项工作。

（八）着力加强社会管理创新，综合治理工作要有新作为。

　　建立健全矛盾纠纷调处体系。落实重大事项社会稳定风险评估机制，从政策层面防止和解决社会矛盾。健全群众工作网络，加强人民调解、行政调解、司法调解工作，组建市仲裁委员会，加强医疗纠纷、道路交通事故等专业性第三方调解组织建设。严格依法处置“两堵两闹”等违法行为。推进劳动保障监察平台建设，切实做好农民工工资支付保障工作，加强劳动安全管理和职业病防治。加快群众来访联合接待服务中心和群众服务平台建设，继续开展领导干部开门接访等活动和积案化解工作，努力解决群众反映强烈的问题。建立健全社会事务管理体系。完善社区建设投入保障机制，加强社区建设，提高社区管理和服务水平。以乡镇、街道综治办和综治工作中心规范化建设为重点，加强综治基层基础建设。完善“四级联动”的工作体制，加快推进流动人口基本公共服务均等化。积极开展帮扶救助关爱行动和安置帮教、社区矫正工作，加强青少年及重点群体管理。积极创建司法所规范化建设示范县（市、区）。扩大法律援助覆盖面。强化网络舆情监控，加强对突发事件和群体性事件的网上舆论引导。发挥工会、共青团、妇联、红十字会等人民团体作用，支持其参与社会管理和公共服务。建立健全社会公共安全体系。完善综合防控体系，继续开展打黑除恶专项行动，严厉打击各类犯罪行为。严格落实安全生产责任制，强化重点行业、重点领域和重点部位的安全监管，加强隐患排查治理，确保安全生产形势持续稳定。以铁的手腕，辅之以一定的经济奖励，上半年要彻底关闭全市所有煤矿。切实加强学校及周边治安环境整治，认真做好中小学生和幼儿交通安全工作。加强食品药品安全日常监管和专项整治。坚持军民融合式发展，巩固深化“三个机制”衔接试点成果，整合应急资源和力量，增强应对突发事件能力。大力支持驻宣部队建设，加强国防教育、国防动员和双拥共建工作。

切实加强政府自身建设

面对发展的新形势、新难题、新要求，政府工作必须坚持做到“四个一”：

坚持“一盘棋”思想

　　实践证明，任何一个地方的发展，都是一个系统工程，其中任何一个环节都不能“停车断档”，否则就会事倍功半，甚至一事无成。全市各级政府及政府各部门必须坚持“守土有责”，多做、早做、做好各自份内的事。特别要自觉维护市委权威，自觉接受市人大、市政协监督，自觉接受各民主党派和工商联的监督，自觉接受群众和舆论监督，真正做到无条件服从服务于宣城的大发展，形成肝胆相照、荣辱与共，齐心协力、干事创业的生动局面。

坚持“一抓到底”

　　做任何工作，做过了不等于落实，只有做好了才是落实。要坚持算着干、现场看、盯着办，具体抓、抓具体。将工作重心、工作精力聚焦到项目上，并实行“挂图式作战”、“一线工作法”、重大项目督查巡查等工作机制，强化绩效管理考核、政务督查和行政问责，把工作盯紧抓实，切实防止工作在层层部署、层层要求中“信号”衰弱甚至中断的问题。只有把“信号”传到位，把工作抓到底，这样的落实才可谓“功夫到家”。

坚持“一刻不放松”

　　抓发展必须抢机遇，抢机遇必须只争朝夕。我们要求发展环境要更加宽松，但环境宽松不等于工作放松，工作放松就等于自我放弃。必须以“慢不得”的危机感、“等不起”的紧迫感、“坐不住”的责任感抓工作，树立雷厉风行、说干就干的作风，认真做好每件事，绝不放松每一天。对确定的事情快办办好、不推不拖不糊，对没有完成的工作要“忍不住、放不下”，再细化工作目标，量化进度指标，倒排工作计划，及时组织实施。

坚持“一身正气”

　　一个人有正气，才能赢得别人尊重；一个干部有正气，才能受到群众拥护。要认真落实党风廉政建设责任制，全面推进廉政风险防控，建立重点项目廉政报告备案等制度，强化审计监督，深化民主考评活动，深入开展工程建设等领域突出问题专项治理，严肃查处违法违纪案件，严肃查处损害群众利益、破坏发展环境的行为。同时，要把事业看重，把得失看轻，把名利看淡，真正做到胸怀坦荡做人、公道正派处事、勤政廉政为民。
